# Dual Sensory Loss and Depressive Symptoms: The Importance of Hearing, Daily Functioning, and Activity Engagement

**DOI:** 10.3389/fnhum.2013.00837

**Published:** 2013-12-16

**Authors:** Kim M. Kiely, Kaarin J. Anstey, Mary A. Luszcz

**Affiliations:** ^1^Centre for Research on Ageing Health and Wellbeing, Australian National University, Canberra, ACT, Australia; ^2^Flinders Centre for Ageing Studies, Flinders University, Adelaide, SA, Australia

**Keywords:** depression, mental health, dual sensory loss, aging, hearing loss, visual impairment, Australian Longitudinal Study of Aging

## Abstract

**Background**: The association between dual sensory loss (DSL) and mental health has been well established. However, most studies have relied on self-report data and lacked measures that would enable researchers to examine causal pathways between DSL and depression. This study seeks to extend this research by examining the effects of DSL on mental health, and identify factors that explain the longitudinal associations between sensory loss and depressive symptoms.

**Methods**: Piecewise linear-mixed models were used to analyze 16-years of longitudinal data collected on up to five occasions from 1611 adults (51% men) aged between 65 and 103 years. Depressive symptoms were assessed by the Centre for Epidemiological Studies Depression (CES-D). Vision loss (VL) was defined by corrected visual acuity >0.3 logMAR in the better eye, blindness, or glaucoma. Hearing loss (HL) was defined by pure-tone average (PTA) >25 dB in the better hearing ear. Analyses were adjusted for socio-demographics, medical conditions, lifestyle behaviors, activities of daily living (ADLs), cognitive function, and social engagement.

**Results**: Unadjusted models indicated that higher levels of depressive symptoms were associated with HL (*B* = 1.16, SE = 0.33) and DSL (*B* = 2.15, SE = 0.39) but not VL. Greater rates of change in depressive symptoms were also evident after the onset of HL (*B* = 0.16, SE = 0.06, *p* < 0.01) and DSL (*B* = 0.30, SE = 0.09, *p* < 0.01). The associations between depressive symptoms and sensory loss were explained by difficulties with ADLs, and social engagement.

**Conclusion**: Vision and HL are highly prevalent among older adults and their co-occurrence may compound their respective impacts on health, functioning, and activity engagement, thereby exerting strong effects on the mental health and wellbeing of those affected. There is therefore a need for rehabilitation programs to be sensitive to the combined effects of sensory loss on individuals.

## Introduction

Sensory loss is a common experience for older adults and is one of the leading causes of non-fatal disease burden for Australians aged 65 years or older (Australian Institute of Health and Welfare, 2007; Kiely et al., 2012b). Hearing loss (HL) in particular is the most prevalent chronic condition affecting the oldest old, yet is often under-recognized and undertreated (Lin, 2012). Age-related declines in sensory functions are gradual and initially unnoticed, but their impacts are substantial and wide ranging. For example, low levels of vision and hearing have been associated with poorer health (Crews and Campbell, 2004) and cognitive function (Lindenberger and Baltes, 1994; Anstey et al., 2003; Lin, 2012), loss of functional independence (Keller et al., 1999; Reuben et al., 1999; Wallhagen et al., 2001; Brennan et al., 2005), communication difficulties (Heine and Browning, 2004), reduced social engagement (Resnick et al., 1997; Crews and Campbell, 2004), poor quality of life (Chia et al., 2006; Fischer et al., 2009), and increased risk of falls (Lopez et al., 2011), hospitalization (Lee et al., 2005), and mortality (Mui et al., 1998; Lee et al., 2007; Karpa et al., 2010). All of these impacts are recognized as risk factors for depression and/or outcomes of depression. Unsurprisingly, a number of studies have consistently demonstrated a strong link between co-occurring vision and HL, often referred to as dual sensory loss (DSL), and depressive symptoms (Lupsakko et al., 2002; Capella-McDonnall, 2005, 2009, 2011a; Chou, 2008; Harada et al., 2008).

Although the association between DSL and mental health is well established, a number of research questions regarding their relationship remain unresolved. These include understanding the direct and indirect mental health consequences in relation to (a) clinical measures of DSL where vision and HL are defined by functional assessment rather than perceived sensory loss; (b) clarifying the relative independent contributions of hearing and vision loss (VL); and (c) identifying mediating factors that explain the association. For example, Capella-McDonnall (2009) has published longitudinal analysis of self-report data from the Health and Retirement Study (HRS) demonstrating that adults who reported VL prior to DSL experienced greater levels of depressive symptoms compared to those who did not report VL, but elevated levels of depressive symptoms were not evident among those who reported HL prior to DSL. Similar findings have also been reported for the English Longitudinal Study of Aging (ELSA), where increased risk of depression incidence was predicted by self-reported VL and DSL but not HL alone (Chou, 2008). It is unclear if DSL has an additional impact on mental health over and above the effects of a single sensory loss (Schneider et al., 2011). Together, these findings support the hypothesis that VL is the primary driver of the association between DSL and depression. However, older adults have a tendency to under report hearing problems in epidemiological surveys (Kiely et al., 2012a). Further, self-report measures may be subject to response endogeneity when examined in relation to depression – that is, adults with mental health problems may hold negative perceptions of their sensory abilities and so be more likely to report difficulties with their vision and hearing. These biases could mask the true nature of the association between the progression of sensory loss and depression. Many studies lack bio-psychosocial data that allow for the investigation of factors that may explain the association between DSL and depression, leaving authors to speculate about potential mechanisms and causal pathways (Brennan and Bally, 2007; Capella-McDonnall, 2009). Addressing these issues is important for the development and implementation of appropriately targeted rehabilitation programs.

This study aims to improve our understanding of the relationship between DSL and mental health by using clinical measures of vision and hearing to examine the long-term impacts of age-related declines in sensory function on depressive symptoms, and to identify mediating bio-psychosocial factors that explain the association. Longitudinal population-based data are used to investigate differences in levels of depressive symptoms between adults with and without sensory loss, and examine discontinuities in rates of change in depressive symptoms after the onset of a single sensory loss and DSL. We employ a similar analytic approach to Capella-McDonnall (2009), whose findings we build upon by defining DSL based on a clinical assessment of vision and hearing. Further, our analyses take into account an extensive range of covariates that have previously been shown to be associated with depression and/or sensory loss and have been hypothesized to be explanatory of the relationship. These covariates include socio-demographics, lifestyle behaviors, medical conditions, functional disability, and activity engagement. Given that HL is under-reported by older adults, and is related to other risk factors for depression, we hypothesize that audiometric HL, as well as VL, will be linked to increased levels of depressive symptoms, and that these associations between sensory loss and depressive symptoms will be explained by activity engagement and functional disability.

## Materials and Methods

### Sample

We report analyses of data from the Australian Longitudinal Study of Aging (ALSA), a representative population-based prospective study of older adults residing in the community and in residential aged care settings (Luszcz et al., 2007). ALSA drew a random sample of adults aged 70 years and older from the electoral roll for the Adelaide metropolitan area of South Australia in 1992 (voting is compulsory). In anticipation of lower response rates and higher mortality-based attrition, ALSA oversampled men aged 85 years and older. Additionally, spouses aged 65 years and older, or adults aged over 70 who were cohabiting with a sampled respondent were also recruited, resulting in a total baseline sample size of 2087 participants.

Data were collected via personal interview and clinical assessment on up to five occasions over a 16-years period (Wave 1 1992; Wave 2, 1994; Wave 3, 2000–2001; Wave 4 2002–2004; Wave 5 2008). The sample analyzed in this study comprised 1611 adults (51% men) aged between 65 and 103 years who completed the clinical assessment on at least one occasion. Due to attrition and wave non-response the panel was unbalanced across waves, and participants who returned to the study after a period of non-participation were retained for the analyses.

### Measures

#### Depressive symptoms

The main outcome of this study, were assessed by the Centre for Epidemiological Studies Depression scale (CES-D; Radloff, 1977). The CES-D is a 20-item instrument that is designed for use in epidemiological surveys to measure depressive symptoms experienced over the past week. Item responses are recorded on a four point Likert scale (0 = none of the time, 1 = some of the time, 2 = quite a bit of the time, 3 = all of the time). Total scores on the CES-D range between 0 and 60, with higher scores indicating greater levels of depressive symptoms and scores >16 reflecting probable depression (Anstey et al., 2007).

#### Sensory variables

Sensory variables included clinical assessment of vision and hearing. VL was defined by corrected distance (3 m) visual acuity >0.3 logMAR (equivalent to 20/40 or 6/12 vision) in the better eye, self-reported blindness, or glaucoma. Hearing was assessed by pure-tone audiometry. A pure-tone average (PTA) was calculated for frequencies of 0.5, 1, 2, and 4 kHz. HL was defined by a PTA >25 dB in the better ear. Participants were categorized into one of four sensory loss groups: no sensory loss (No SL), VL only, HL only, and DSL. Time was defined as years in study, and three variables reflecting time after the onset of sensory loss (post-time VL, post-time HL, and post-time DSL) were also created. These variables were used to assess differences in rates of change in CES-D scores before and after the onset of sensory loss.

#### Covariates

Socio-demographic variables included age, sex, education, marital status, and domicile. Age at baseline was mean centered at 78 years (range: 65–103). Education was defined by age left school and was mean centered at age 15. Marital status was coded as partnered (married or *de facto*), un-partnered (never married, separated), and widowed. Participants were classified as either living in the community (private residence) or in residential care (e.g., hostels, nursing homes, hospitals, or boarding houses). Life style factors included smoking status and alcohol use. Participants were classified as never smokers, former smokers, and current smokers. The coding of alcohol consumption was in line with current Australian National Health and Medical Research Council guidelines (NHMRC, 2009), and was defined by the average number of standard drinks consumed in a single session (abstain, two or fewer standard drinks, and more than two standard drinks). Medical conditions were obtained by self-report of clinician diagnoses and included: arthritis, hypertension, diabetes, cardiovascular disease, history of stroke, and cancer. Cognitive impairment was assessed by the Mini Mental State Examination (MMSE) (Folstein et al., 1975).

Disability and daily functioning were defined by the number of reported difficulties (no difficulty, any difficulty) with activities of daily living (ADLS) and instrumental activities of daily living (IADLs). ADLs included the following eight basic activities important for daily functioning: grooming, dressing, eating, bathing, toileting, moving inside the home, and transferring from bed to chair. IADLs included an additional 10 routine tasks: doing the laundry, light housework, heavy housework, home maintenance or gardening, meal preparation, using the telephone, managing money, using public transport, grocery shopping, and writing a letter. The number of difficulties with ADLs and IADLs were coded to reflect no difficulties, one difficulty, two difficulties, and three or more difficulties.

The Adelaide activity profile (AAP) was used to assess level of social and activity engagement. The AAP has been validated as a measure of lifestyle activities for older adults for use in epidemiological surveys (Clark and Bond, 1995; Bond and Clark, 1998; Isherwood et al., 2012). Social activity engagement was defined by responses to eight items requiring participants to report their level of participation in social activities over a 3-month period. Items included: attending religious services, outdoor social activities, organized social activities at a club or center, making telephone calls to friends and family, inviting people to visit at home, going on a drive or outing, performing voluntary or paid work. A measure of engagement in mentally stimulating activities was calculated based on levels of participation with the following four activities: spending time on a hobby, reading, watching TV or listening to the radio, and walking outside for 15 min or more. Higher scores reflected a greater level of engagement and scores were mean centered. A measure reflecting the specific role of HL in restricting social engagement was also included. Participants were asked “does hearing affect your personal or social life?” with responses coded on a four point scale (0 = never, 1 = seldom, 2 = sometimes, 3 = often).

All variables were time-varying and collected at each measurement occasion, with the exception of age at baseline, sex, and education.

### Analyses

In line with standard model building procedures for testing discontinuities in intercepts and change as described by Singer and Willett (2003), we conducted a series of piecewise linear-mixed models to test for the longitudinal effects of sensory loss on depressive symptoms over time. In an initial step we tested an unconditional means model (an intercept only model not adjusted for any covariates) and unconditional growth model (adjusted only for time in study), which served as base comparison models for subsequent analyses. We then added to the unconditional growth model fixed and random effects for the three dummy-coded indicators of sensory loss (VL, HL, and DSL), and the three post-time sensory loss variables (time-post VL, time-post HL, and time-post DSL). The purpose of this model was to assess discontinuities in levels and rates of change in CES-D scores as a function of sensory loss. Specifically, the time-varying indicators of sensory loss reflect differences in levels of depressive symptoms between participants with a sensory loss relative to those with no sensory loss. The time in study variable reflects rates of change in depressive symptoms for participants with no sensory loss (or prior to the first observed instance of sensory loss). The three post-time sensory loss variables reflect differences in linear rates of change in depressive symptoms for participants during times that they are experiencing sensory loss, relative to participants who are not experiencing a sensory loss. The level 1 (individual level) and level 2 (population average level) equations tested for the sensory loss adjusted piecewise linear-mixed model with fixed and random effects for all variables are:

Level 1:
  $$\begin{array}{llll} \mathrm{CES}D_{i,j} & =\pi_{0i}+\pi_{1i}\left(\mathrm{Tim}e_{i,j}\right)+\pi_{2i}\left(VL_{i,j}\right)+\pi_{3i}\left(HL_{i,j}\right)\; & & \;\\ & \;+\pi_{4i}\left(\mathrm{DS}L_{i,j}\right)+\pi_{5i}\left(\mathrm{Time post V}L_{i,j}\right)\; & & \;\\ & \;+\pi_{6i}\left(\mathrm{Time post H}L_{i,j}\right)+\pi_{7i}\left(\mathrm{Time post DS}L_{i,j}\right)\; & & \;\\ & \;+\varepsilon_{i,j}\; & & \; \end{array}$$
Level 2:
  $$\begin{array}{llll} & \pi_{\mathrm{0i}}=\beta_{00}+U_{0i}\;\left(\mathrm{Intercept}\right)\; & & \;\\ & \pi_{\mathrm{1i}}=\beta_{10}+U_{1i}\;\left(\mathrm{Time}\right)\; & & \;\\ & \pi_{\mathrm{2i}}=\beta_{20}+U_{2i}\;\left(\mathrm{VL}\right)\; & & \;\\ & \pi_{\mathrm{3i}}=\beta_{30}+U_{3i}\;\left(\mathrm{HL}\right)\; & & \;\\ & \pi_{\mathrm{4i}}=\beta_{40}+U_{4i}\;\left(\mathrm{DSL}\right)\; & & \;\\ & \pi_{\mathrm{5i}}=\beta_{50}+U_{5i}\;\left(\mathrm{TimepostVL}\right)\; & & \;\\ & \pi_{\mathrm{6i}}=\beta_{60}+U_{6i}\;\left(\mathrm{TimepostHL}\right)\; & & \;\\ & \pi_{\mathrm{7i}}=\beta_{70}+U_{7i}\;\left(\mathrm{TimepostDSL}\right)\; & & \; \end{array}$$

Where, π represents the estimated CES-D score for a given time-varying covariate for person *i*, and ε represents the level 1 residual (unexplained variance). In the level 2 equation β represents the population average (fixed effect) association between a given variable and CES-D score, and *U* represents the population average (random effect) variance component for a given variable. Akaike information criterion (AIC) and Bayesian information criterion (BIC) indices tests of Chi square differences were used to assess the optimal model fit relative to the unconditional means and unconditional growth model. To reduce model complexity and facilitate model convergence, random effects that did not contribute to the overall model fit were excluded from subsequent multivariate adjusted models.

To identify explanatory variables that attenuate the longitudinal associations between sensory loss and depression, two series of analyses were conducted. First, covariates were added individually to age, sex, and education adjusted models. These analyses tested the extent to which a single variable set explained the association between sensory loss and CES-D scores without taking into consideration the shared effects of other factors. Second, to assess how each variable set explained the association independently of the effects of other factors, each set of variables were added sequentially to the optimal sensory loss adjusted model in the following sequence:

- Model 1 added time invariant socio-demographic variables (e.g., age at baseline, sex, education).
- Model 2 added time-varying socio-demographic variables reflecting participants’ current circumstances (marital status and domicile).
- Model 3 added life style behaviors (e.g., smoking and alcohol consumption).
- Model 4 added medical conditions (e.g., diabetes, cardiovascular disease).
- Model 5 added cognitive function (MMSE).
- Model 6 added markers of disability (e.g., ADLs and IADLs).
- Model 7 added activity engagement variables (social engagement, mental engagement, hearing related social limitations).

There were 1550 participants with complete data on all covariates, representing a loss of 4% of the available sample (*n* = 1611) due to item non-response. We therefore employed multiple imputation to reduce bias attributable to this item level missing data (Schafer and Graham, 2002; Graham et al., 2007; Graham, 2009; Janssen et al., 2010). We imputed 20 datasets under the missing at random (MAR) assumption which maintains that after controlling for observed variables, the remaining missing data is not associated with any unobserved factors and can be considered completely random. The imputation model included all variables from the full analytic model, plus auxiliary variables (self-rated health, country of origin, preferred language, career occupation, PTA, visual acuity, and individual scale items). Data were not imputed for participants who had missing data for an entire wave due to attrition or mortality. Results from the multiple imputation analyses were compared to complete case analyses.

To illustrate the effects of sensory loss progression on depressive symptoms estimates from the sensory loss adjusted model were used to graph the predicted trajectories in CES-D scores over 16-years for four hypothetical cases who experienced: (i) no SL at any time, (ii) VL from year 4, (iii) HL from year 4, (iv) DSL from year 7. All analyses were conducted using Stata 11 statistical software (StataCorp, 2009).

## Results

The baseline sample characteristics for each sensory loss group are presented in Table 1. There were 1246 participants (mean age = 78) with data on sensory loss and depressive symptoms at baseline. A number of participants who did not complete the clinical assessment at baseline did so at later waves, resulting in a total sample size of 1611 participants. Sensory loss was highly prevalent, particularly among the oldest old. Only 10.5% of participants aged 85–94 were identified with no sensory loss, and all participants aged 95 years and older experienced some degree of sensory loss. Tests of bivariate associations indicated that adults with DSL were more likely to be male, older, widowed, residing in residential care settings, have lower levels of education, abstain from alcohol consumption, rate their health as fair or poor, have been diagnosed with diabetes and experience a greater number of difficulties with ADLs and IADLs. In contrast, single sensory loss did not show strong discrimination for many of these variables. HL was more prevalent among men than women, and increased slightly with age, while the prevalence of VL actually decreased among older age-groups reflecting the inflow of older participants to DSL. Over the course of the study, on average 21.1% of the sample had no sensory loss, 6.5% had VL only, 47.2% had HL only, and 25.2% had DSL. A total of 584 participants were identified with DSL on at least one occasion. For the majority of incident DSL cases, onset of HL preceded VL. Attrition and mortality were not the only sources of missing data, there were 373 (23%) participants who completed the clinical assessment of sensory functioning and CES-D but had item level missing data for other covariates selected for inclusion in the multivariate analyses. The means and standard deviations in CES-D scores by loss group at each measurement wave are presented in Table 2. The intra-class correlation for CES-D scores was 0.49 (standard error = 0.02), indicating that roughly equal proportions of the unexplained variance could be attributed to between and within person differences.

**Table 1:** **Sample characteristics at baseline by sensory loss (*N* = 1252^a^)**.

	Whole sample *n*	No sensory loss %	Vision loss only %	Hearing loss only %	Dual sensory loss %	Test statistic, *p*-value
Sex
Men	636	19.7	6.1	49.8	24.4	χ^2^ = 25.8, *p* < 0.001
Women	616	30.7	8.1	43.2	18.0	
Age group
65–74	91	51.6	9.9	33.0	5.5	χ^2^ = 179.6, *p* < 0.001
75–84	702	31.9	7.5	46.9	13.7	
85–94	415	10.4	6.3	49.6	33.7	
95+	44	0.0	2.3	40.9	56.8	
Marital status
Partnered	857	30.0	5.3	51.0	13.8	χ^2^ = 29.9, *p* < 0.001
Un-partnered	66	28.8	7.6	47.0	16.7	
Widowed	309	16.8	6.5	48.9	27.8	
Residence
Community	1196	25.9	7.2	46.8	20.1	χ^2^ = 25.6, *p* < 0.001
Institution	56	7.1	5.4	41.1	46.4	
Age left school
≤14	627	20.6	5.9	47.4	26.2	χ^2^ = 13.6, *p* = 0.003
>14	533	25.9	8.1	47.8	18.2	
Country of birth
Australia	859	24.6	6.9	47.6	21.0	χ^2^ = 0.6, *p* = 0.444
Overseas	393	26.2	7.6	44.3	21.9	
Hearing aid use
Yes	183	1.6	0.6	66.7	31.2	χ^2^ = 88.6, *p* < 0.001
No	1068	29.1	8.2	43.2	19.5	
Smoking status
Never	600	25.5	8.0	46.8	19.7	χ^2^ = 3.8, *p* = 0.702
Former	559	25.0	5.9	46.2	22.9	
Current	90	23.3	8.9	46.7	21.1	
Alcohol consumption
Abstain	443	20.1	7.9	47.9	24.2	χ^2^ = 13.0, *p* < 0.043
1–2 Standard drinks	645	28.1	6.0	45.7	20.2	
3+ Standard drinks	161	27.3	9.3	46.0	17.4	
Medical conditions
Arthritis	652	26.2	7.2	48.0	18.6	χ^2^ = 5.9, *p* = 0.113
Hypertension	316	29.7	6.6	42.7	20.9	χ^2^ = 5.2, *p* = 0.160
Diabetes	82	14.6	7.3	39.0	39.0	χ^2^ = 17.9, *p* < 0.001
CVD	227	26.0	6.2	44.5	23.3	χ^2^ = 1.3 *p* = 0.736
Stroke	47	12.8	8.5	46.8	31.9	χ^2^ = 5.7, *p* = 0.127
Self-rated health
Excellent/very good	482	25.7	6.2	50.2	17.8	χ^2^ = 15.9, *p* = 0.014
Good	391	28.9	7.2	43.0	21.0	
Fair/poor	379	20.3	8.2	45.6	25.9	
ADL difficulties
None	1063	27.2	6.5	47.3	19.0	χ^2^ = 40.4, *p* < 0.001
One	88	13.6	12.5	45.5	28.4	
Two	45	15.6	13.3	35.6	35.6	
Three +	56	10.7	5.4	42.9	41.1	
IADL difficulties
None	822	25.9	7.1	47.6	19.5	χ^2^ = 22.6, *p* = 0.007
One	197	24.9	7.1	46.2	21.8	
Two	97	27.8	8.2	49.5	14.4	
Three +	136	18.4	6.6	39.0	36.0	

*Vision loss only defined by corrected visual acuity >0.3 logMAR in the better eye, blindness, or glaucoma (with no co-occurring hearing loss)*.

*Hearing loss only defined by PTA >25 dB in the better ear (with no co-occurring vision loss)*.

*Dual sensory loss defined by co-occurring hearing and vision loss*.

*CVD: cardio vascular disease*.

*ADL: activities of daily living (bathing, grooming, dressing, eating, toileting, bed to chair transfer, difficulty inside home, difficulty away from home)*.

*IADL: instrumental activities of daily living (laundry, light housework, heavy housework, home maintenance, preparing meals, using the telephone, managing money, shopping, using public transport, writing letters)*.

*^a^ Includes six participants without baseline CES-D data*.

**Table 2:** **Frequency counts and CES-D means and standard deviations (SD) for each sensory loss group by wave**.

	No sensory loss			Vision loss only			Hearing loss only			Dual sensory loss			Overall		
		CES-D			CES-D			CES-D			CES-D			CES-D	
	*n*	Mean	SD	*n*	Mean	SD	*n*	Mean	SD	*n*	Mean	SD	*n*	Mean	SD
Wave 1	314	6.96	6.60	89	7.21	5.48	580	8.38	7.22	263	9.19	8.22	1246	8.11	7.23
Wave 2	277	7.01	6.25	61	7.20	6.77	560	7.95	7.23	259	10.10	7.71	1157	8.16	7.17
Wave 3	75	7.87	6.28	33	8.00	5.82	212	9.34	7.33	109	9.55	7.62	429	9.03	7.13
Wave 4	36	7.89	6.76	25	9.00	7.96	137	8.40	7.35	89	9.22	7.89	287	8.64	7.48
Wave 5	8	7.88	5.84	8	7.49	5.60	75	10.7	7.31	34	12.00	9.00	125	10.60	7.66

*Panel data is unbalanced, and participants who leave and return to study were retained for analyses (*N* = 1611)*.

*Vision loss defined by corrected visual acuity >0.3 logMAR in the better eye, blindness, or glaucoma*.

*Hearing loss defined by PTA >25 dB in the better ear*.

*Dual sensory loss defined by co-occurring hearing and vision loss*.

A series of models were tested to identify the optimal parameterization relative to the unconditional growth model (Table 3). Residual plots confirmed that the assumptions of linear regression were met, and transformations of CESD score did not alter the substantive findings. Broadly, the inclusion of sensory loss variables as fixed effects improved model fit (Model A). The addition of random effects for DSL and time-post DSL made further substantial improvements in model fit (Model C) while the addition of random effects for HL and time-post HL made a small but significant improvement in model fit (Model D). Thus, the effects of HL and DSL on levels and rates of change in CES-D scores varied across individuals. Although the fit indices indicated that the inclusion of VL did not contribute to the overall model fit, this variable was retained in subsequent analyses to distinguish this group from participants with no sensory loss.

**Table 3:** **Model fit indices for competing models piecewise linear-mixed models of change in CES-D scores as a function of sensory loss (*n* = 1611)**.

Model	AIC	Δχ2	Δdf	*p*	Comparison model
Intercept only (IO)	22226				
Unconditional growth (UG)	22132	99.80	3	<0.001	IO
Sensory adjusted model A	22097	42.90	4	<0.001	UG
Sensory adjusted model B	22099	0.52	1	0.471	Model A
Sensory adjusted model C	22081	19.68	2	<0.001	Model A
Sensory adjusted model D	22079	6.08	2	0.048	Model C

*$\sigma_{\varepsilon }^{2}$: Level 1 residual; AIC: Akaike information criterion*.

*Model A: UG+ fixed effects (VL, HL, DSL, time-post HL, time-post DSL)*.

*Model B: Model A+ fixed effects (time-post VL)*.

*Model C: Model A+ random effects (DSL, time-post DSL)*.

*Model D: Model C+ random effects (HL, time-post HL), best fitting model*.

**Figure 1:**
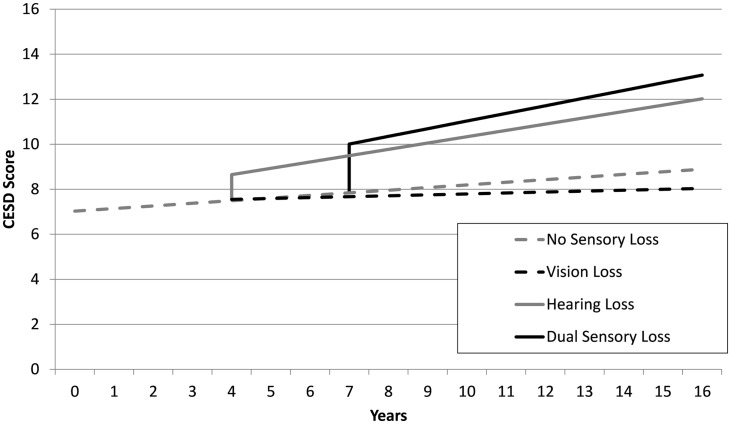
**Mean trajectories estimated from unadjusted linear-mixed models, depicting discontinuities in levels and rates of change of CES-D scores as a function of sensory loss for four hypothetical scenarios: no sensory loss at any time (gray dash), vision loss only from year 4 (black dash), hearing loss only from year 4 (gray line), dual sensory loss from year 7 (black line)**.

The estimates from sensory loss adjusted analysis indicated that participants identified with DSL (β = 2.17, SE = 0.39, *p* < 0.001) and HL only (β = 1.14, SE = 0.32, *p* < 0.001) had elevated levels of depressive symptoms compared to those with no sensory loss. Participants with DSL had significantly higher levels of CES-D scores compared to those with HL (Mean Difference = 1.03, SE = 0.34, *p* = 0.003). However, there was no reliable difference in levels of CES-D scores between those identified with VL only and those identified with no sensory loss (*p* = 0.91). There was an estimated annual rate of change in CES-D scores of 0.12 (SE = 0.04, *p* = 0.004) units per year among adults not experiencing a sensory loss. An additional increase in linear rates of change in CES-D scores occurred after the first instance of HL (β = 0.17, SE = 0.06, *p* = 0.006) and DSL (β = 0.23, SE = 0.09, *p* < 0.001). There was no difference in rates of change in CES-D scores after the first instance of VL (*p* = 0.47) relative to adults with no sensory loss. Figure 1 provides a graphical illustration of these differences in levels and rates of change in CES-D scores before and after the first occurrence that a sensory loss was observed. There was also an association between the degree of HL and depressive symptoms scores, with higher PTA thresholds predicting higher CESD scores (results not tabled).

To assess the extent to which the associations between sensory loss and CESD-D scores were explained by the effects of other factors, covariates were first added individually to age, sex, and education adjusted Model D (Table 4) and then in a series of hierarchical steps (Table 5). After adjusting for activity engagement, none of the sensory loss variables (DSL, HL, VL, time-post DSL, time-post HL) reliably predicted differences in levels or rates of change in CES-D scores. The associations between sensory loss and CES-D were also attenuated after the inclusion of ADLs and IADLs.

**Table 4:** **Parameter estimates from piecewise linear-mixed models testing predictors of CES-D scores in Model G, adjusted for covariates, from 20 multiply imputed datasets (*n* = 1611)**.

	Model 1		Model 1 + marital status and residence		Model 1 + lifestyle		Model 1 + medical conditions		Model 1 + MMSE		Model 1 + ADLs and IADLs		Model 1 + engagement	
	Est.	SE	Est.	SE	Est.	SE	Est.	SE	Est.	SE	Est.	SE	Est.	SE
**FIXED EFFECTS**														
Intercept	6.87***	0.33	6.53***	0.34	6.09***	0.43	5.92***	0.36	7.17***	0.34	6.11***	0.33	6.62***	0.33
Sensory loss (no sensory loss)
Dual sensory loss	1.58***	0.43	1.53***	0.42	1.46***	0.42	1.49***	0.42	1.45***	0.42	1.30**	0.42	0.82	0.42
Hearing loss only	0.85**	0.33	0.88**	0.33	0.79*	0.33	0.78*	0.32	0.78*	0.33	0.69*	0.32	0.49	0.32
Vision loss only	−0.07	0.48	−0.03	0.48	− 0.1	0.48	− 0.08	0.48	− 0.15	0.48	− 0.34	0.48	− 0.2	0.48
Time
Years in study	0.14***	0.04	0.10**	0.04	0.15***	0.04	0.09*	0.04	0.10*	0.04	0.04	0.04	0.13***	0.04
Time-post DSL onset	0.20*	0.09	0.19*	0.09	0.18*	0.09	0.16	0.08	0.20*	0.09	0.17	0.09	0.14	0.09
Time-post HL onset	0.17**	0.06	0.16**	0.06	0.16**	0.06	0.16**	0.06	0.17**	0.06	0.14*	0.06	0.11	0.06
Time-post VL onset														
Age at baseline	0.13***	0.03	0.09**	0.03	0.14***	0.03	0.12***	0.03	0.11***	0.03	0.08**	0.03	0.07*	0.03
Sex (men)
Women	1.06**	0.32	0.81*	0.33	1.32***	0.36	0.96**	0.32	0.94**	0.32	0.69*	0.31	1.34***	0.31
Education (age 15)
Age left school	−0.42***	0.12	−0.40***	0.12	− 0.41***	0.12	− 0.40***	0.11	− 0.33**	0.12	− 0.41***	0.11	− 0.31**	0.11
Marital status (partnered)
Un-partnered			0.54	0.66										
Widowed			1.04***	0.31										
Residence (community)
Institution			1.20**	0.43										
Smoking status (never)
Former smoker					0.81*	0.36								
Current smoker					2.47***	0.62								
Alcohol consumption (no risk)
Abstain					0.57*	0.29								
Risky, >2 standard drinks					− 0.05	0.42								
Medical conditions
Arthritis							1.20***	0.25						
Hypertension							0.02	0.29						
Diabetes							− 0.03	0.48						
CVD							1.80***	0.30						
Stroke							2.19***	0.49						
Cancer							1.57**	0.57						
Cognitive impairment
MMSE									− 0.20***	0.04				
Difficulties with IADLs (none)														
One IADL difficulty											0.98***	0.28		
Two IADL difficulties											1.62***	0.38		
Three or more IADL difficulties											1.93***	0.33		
Difficulties with ADLs (none)														
One ADL difficulty											1.66***	0.35		
Two ADL difficulties											1.81***	0.48		
Three or more ADL difficulties											2.79***	0.33		
Activity engagement
Social activity profile													− 0.22***	0.04
Mental activity profile													− 0.34***	0.05
Restricts social life (never)														
Seldom													0.4	0.43
Sometimes													2.02***	0.37
Often													2.67***	0.56
**RANDOM EFFECTS**														
Variance
Intercept	23.59		22.91		22.94		21.71		22.34		19.72		20.75	
Time	0.02		0.02		0.02		0.01		0.02		0.02		0.01	
Time-post DSL	0.16		16.57		0.17		0.13		0.15		0.19		0.19	
Time-post HL	0.10		0.09		0.11		0.11		0.10		0.06		0.09	
DSL	12.47		12.36		12.18		13.06		12.89		11.71		10.80	
HL	3.78		3.69		4.37		4.09		3.85		3.28		3.10	
Residual	22.25		22.37		22.17		22.05		22.41		22.85		22.42	

*p < 0.05, **p < 0.01, ***p < 0.001.

*SE: standard error*.

*CVD: cardio vascular disease*.

*ADL: activities of daily living (bathing, grooming, dressing, eating, toileting, bed to chair transfer, difficulty inside home, difficulty away from home)*.

*IADL: instrumental activities of daily living (laundry, light housework, heavy housework, home maintenance, preparing meals, using the telephone, managing money, shopping, using public transport, writing letters)*.

**Table 5:** **Parameter estimates from piecewise linear-mixed models testing predictors of CES-D score, from 20 multiply imputed datasets (*n* = 1611)**.

	Unadjusted		Model 5		Model 6		Model 7	
	Est.	SE	Est.	SE	Est.	SE	Est.	SE
**FIXED EFFECTS**								
Intercept	7.01***	0.28	5.16***	0.44	4.68***	0.43	4.44***	0.43
Sensory loss (no sensory loss)
Dual sensory loss	2.17***	0.41	1.20**	0.42	0.96*	0.41	0.37	0.41
Hearing loss only	1.14***	0.32	0.70*	0.32	0.57	0.31	0.29	0.31
Vision loss only	0.07	0.48	−0.14	0.47	−0.39	0.47	−0.43	0.46
Time
Years in study	0.12**	0.04	0.02	0.04	−0.06	0.04	−0.05	0.04
Time-post DSL onset	0.23*	0.09	0.14	0.08	0.13	0.09	0.08	0.09
Time-post HL onset	0.17**	0.06	0.15*	0.06	0.13*	0.06	0.09	0.06
Time-post VL onset	−0.09	0.10						
Age at baseline			0.08**	0.03	0.03	0.03	−0.02	0.03
Sex (men)
Women			0.87*	0.35	0.56	0.34	0.86**	0.33
Education (age 15)
Age left school			−0.30**	0.11	−0.29**	0.11	−0.23*	0.11
Marital status (partnered)
Un-partnered			0.49	0.63	0.35	0.61	0.4	0.6
Widowed			0.99**	0.30	0.91**	0.29	1.12***	0.29
Residence (community)
Institution			1.06*	0.42	0.93*	0.42	0.99*	0.42
Smoking status (never)
Former smoker			0.80*	0.35	0.79*	0.33	0.87**	0.33
Current smoker			2.53***	0.60	2.43***	0.58	2.24***	0.57
Alcohol consumption (no risk)
Abstain			0.44	0.28	0.41	0.28	0.29	0.27
Risky, >2 standard drinks			−0.11	0.41	−0.11	0.40	−0.07	0.4
Medical conditions
Arthritis			1.17***	0.25	1.05***	0.24	0.94***	0.24
Hypertension			0.13	0.29	0.05	0.28	0.12	0.28
Diabetes			−0.11	0.48	−0.14	0.47	−0.05	0.47
CVD			1.89***	0.29	1.68***	0.29	1.60***	0.29
Stroke			2.12***	0.49	1.55**	0.49	1.43**	0.48
Cancer			1.70**	0.57	1.54**	0.57	1.56**	0.57
Cognitive impairment
MMSE			−0.20***	0.04	−0.22***	0.04	−0.18***	0.04
Difficulties with IADLs (none)
One IADL difficulty					0.88**	0.28	0.84**	0.28
Two IADL difficulties					1.41***	0.37	1.38***	0.37
Three or more IADL difficulties					1.86***	0.33	1.82***	0.32
Difficulties with ADLs (none)
One ADL difficulty					1.41***	0.35	1.25***	0.35
Two ADL difficulties					1.40**	0.48	1.14*	0.48
Three or more ADL difficulties					2.62***	0.33	2.13***	0.33
Activity engagement
Social activity profile							−0.20***	0.03
Mental activity profile							−0.26***	0.05
Hearing restricts social life (never)
Seldom							0.33	0.42
Sometimes							1.79***	0.37
Often							2.39***	0.55

*p < 0.05, **p < 0.01, ***p < 0.001.

## Discussion

Previous research has established a link between perceived sensory loss and poor mental health, though combined VL and HL has not consistently been reported to have additional burden over and above the effects of a single sensory loss (Capella-McDonnall, 2009; Schneider et al., 2011). Understanding the pathways between DSL and depressive symptoms in late-life is important for the health management and care of older adults. However, no studies have examined long-term changes in depressive symptoms in relation to the progression of vision and HL using clinical measures of sensory function, while at the same time taking into account a rich set of contextual and potentially mediating factors.

Regarding incident sensory loss, the present findings illustrated that the first observed occurrence of HL and DSL coincided with a jump in the level of depressive symptoms. In contrast, no increase in the level of depressive symptoms was observed at the first observed occurrence of VL. Notably, the elevation in depressive symptoms associated with DSL was almost twice that of HL indicating that co-occurring problems with vision and hearing contribute additional burden to mental health over and above the effects of a single sensory loss. There was also a greater increase in depressive symptoms over time after the onset of DSL and HL, compared to adults with VL or no sensory loss. Consistent with previous studies (Capella-McDonnall, 2009) we observed considerable variability between individuals in the way that the progression of DSL impacted on mental health profiles.

Overall, we found that impaired visual functioning was not associated with poorer mental health, except when combined with HL. On this evidence, audiometric HL appears to be the main driver of the association between clinically defined DSL and increased depressive symptoms. This contrasts with previous studies that have used self-report data and identified perceived VL, rather than perceived HL, as being more strongly associated with increased risk of depression or greater propensity to report depressive symptoms (Crews and Campbell, 2004; Capella-McDonnall, 2005, 2009; Chou, 2008). Crews and Campbell (2004) also found that adults who report VL have a greater number of difficulties with ADLs than do adults who report HL. These discrepancies with our findings most likely reflect differences in the assessment, measurement, and definition of sensory loss, demonstrating that self-report measures operate differently to clinical measures. Further, adults with severe VL and specific eye disease such as age-related macular degeneration have been consistently reported to be at increased risk of depression (Brody et al., 2001; Casten et al., 2004; Evans et al., 2007; Popescu et al., 2012; Eramudugolla et al., 2013). This literature may also seem at odds with the present findings. However, these studies investigated vision in isolation, and did not include measures of hearing function. Our null finding only applies to VL in the absence of HL. Given the high prevalence of HL among older adults, there were relatively few adults with “VL only” in our study and they tended to be younger. Moreover, recent evidence on the association between visual acuity (a main component of our VL variable) and depression has been mixed. Eramudugolla et al. (2013) reported an association between depression and low contrast visual acuity but not high contrast visual acuity, and in a nationally representative survey of US adults Zhang et al. (2013) found that self-reported VL independently predicted increased depression risk, but visual acuity did not.

A key outcome of this study was the identification of explanatory factors that attenuate the association between sensory loss and depressive symptoms, and can be targeted by interventions. Virtually all the variables examined weakened the relationship. Most notably, participation levels in socially engaging and mentally stimulating activities fully explained the increased depressive symptoms experienced by adults with sensory loss. The next strongest mediators of the association were difficulties with daily functioning. This evidence supports the notion that maintaining an active and engaged lifestyle by participating in meaningful activities can mitigate the adverse impacts caused by functional limitations and frailty, and is important for older adults’ health and wellbeing. Capella-McDonnall (2011a) hypothesizes that increased dependence on others and greater need for assistance due to age-related sensory loss can lead to an externalized shift in locus of control and poorer self-concept. Thus, older adults may be at increased risk of depression if they do not have sufficient resources available to cope with the restricted independence and communication difficulties caused by sensory declines. Future research should investigate the role of personal resources such as perceived control.

Previous studies investigating mediators of perceived sensory loss and mental health have been mixed. Cross-sectional analysis of community-dwelling adults in the USA found that self-reported sensory loss reliably predicted depressive symptoms independently of markers of socio-economic position, health, social support, activity engagement, and disability (Capella-McDonnall, 2005). On the other hand, Chou (2008) reported that after adjusting for mobility impairment, informal support and limitations with daily functioning, VL remained an independent predictor of depression risk but HL and DSL did not. Not only do these cross-sectional findings conflict with each other, neither pattern is consistent with our longitudinal findings. This may reflect the differences of self-reports compared to clinical measures of function, or the choice of covariates.

Other longitudinal studies, though based on self-report data, have provided findings more in line with ours. For example, volunteer programs were shown to provide protective effects against depression for adults with self-reported DSL (Capella-McDonnall, 2011a) and physical status has been identified as a moderator of this association (Capella-McDonnall, 2011b). Similar analyses have also shown that adults adapt to their sensory loss and after a period of adjustment experience an improvement in their level of depressive symptoms. (Capella-McDonnall, 2009). Unfortunately the small number of repeated observations (*t* = 5) and high proportion of missing data at later waves precluded modeling of quadratic slopes after the onset of sensory loss in our study. This would have facilitated testing of adaption to sensory loss. The selection, optimization, and compensation model (Baltes et al., 1999) and assimilative and accommodative coping model (Rothermund and Brandtstadter, 2003) are useful frameworks for conceptualizing successful adaption to sensory loss as they emphasize the importance of shifting personal goals and active interventions in response to loss. Factors hypothesized to enable the adaption process would include cognitive resources (Heyl and Wahl, 2011), sense of control, use of sensory aids, social and environmental support, and changes in expectations (Brennan and Bally, 2007).

As outlined, most of the research on DSL has been primarily reliant on epidemiological survey data that has been used to provide descriptive population estimates derived from self-report measures (Brennan and Bally, 2007; Schneider et al., 2011), which are argued to be more ecologically valid, providing an assessment of the perceived impact of sensory loss (Heine et al., 2013). While analyses of self-report data make an important contribution to our understanding of how perceptions of sensory loss relate to healthy aging, they should not be conflated with processes associated with functional abilities. Self-report data has limited validity as a measure of impairment as perceptions of sensory loss are influenced by individual differences in health expectations – which are shaped by social norms (Sargent-Cox et al., 2008). Because declining hearing is a common experience in late life, many older adults perceive hearing difficulties to be a normal part of the aging process and so are less likely to report a HL, even in severe cases (Kiely et al., 2012a). Further, adults with poor mental health are more likely to report difficulties with vision and hearing, which introduces response bias and makes it difficult to draw clear conclusions regarding the direction of the association between self-reported DSL and depressive symptoms. We found that the association between DSL and depression was partly explained by self-reports that hearing limitations restricted a person’s social life. This along with the finding of Zhang et al. (2013) could be interpreted as showing that self-reported sensory loss is more relevant when investigating their impacts on wellbeing. By using clinical measures we were able to ascertain factors that underlie the association between sensory impairment and depressive symptoms, and therefore identify circumstances that might lead to reports of difficulties with sensory functioning.

There is no standard or clear definition of DSL (Saunders and Echt, 2007; Smith et al., 2008). While we applied cut-points for mild impaired vision (visual acuity >0.3 logMAR) and hearing (PTA >25 dB in the better hearing ear) that conformed to common international standards, it is possible that when defining DSL the ideal thresholds may change depending on the interaction between sensory modalities. We also recognize that VL and HL occur along a continuum and the common practice of defining ranges of sensory impairment based on arbitrary, though conventional, thresholds overlooks the complex interactions that occur between different sensory domains and may conceal some effects.

Limitations of this study include biases introduced by attrition and non-response, diminished sample size at later waves, and lack of clinical assessment of eye disease such as age-related macular degeneration that have previously been linked to depression. Despite the inclusion of adults residing in institutions and oversampling of older men, the ALSA is still subject to selective attrition due to poor health which could introduce biases (Anstey and Luszcz, 2002). Although ALSA interviewers were trained to collect data from older adults with functional limitations, severe loss of vision and hearing are still likely to hinder survey completion. Consequently, it is likely that our sample represents a healthy population of older adults and estimates on the prevalence and impact of sensory loss should be considered conservative.

Due to the long time intervals between measurement occasions it was not possible to precisely model the timing of sensory loss onset, so the first observed occurrence of a sensory loss can only be interpreted to indicate that visual acuity or hearing function crossed a threshold commonly used to define mild impairment at some time in the year(s) between measurement occasions. It is likely that the progression of sensory loss will differ across individuals and could occur suddenly or unfold gradually over time. Such complexities could not be captured in the present analyses. Moreover, we did not examine the severity of sensory loss and used basic identifiers of impaired visual and auditory function. VL was defined by impaired visual acuity or presence of glaucoma and blindness. Other aspects of vision that were not assessed in this study, such as poor contrast sensitivity, reduced peripheral vision, and macular degeneration, could be more important for physical functioning and mental health. Similarly, our use of PTA in the better hearing ear disregards much of the richness of data available in an audiogram and we lacked measures of central auditory functioning. Although we have replaced a poor marker of HL (self-reported hearing difficulties) with a more ideal measure of hearing function (PTA >25 dB), it is possible that at the same time we have applied a narrower definition of VL (visual acuity >0.3 logMAR and eye disease) that does not adequately reflect broader aspects of visual functioning that would otherwise be captured by self-report. Future research would extend existing findings by investigating other measures of hearing and vision, such as speech recognition, peripheral vision, and contrast sensitivity (e.g., Fischer et al., 2009). This would enable a rigorous definition of DSL, as well as allowing for greater sophistication in our understanding of the co-morbidity of vision and hearing impairment and associated functional limitations. These limitations notwithstanding, the strengths of this study are the use of population-based longitudinal data, use of multiple imputation, clinical measures of sensory loss, and the inclusion of an extensive range of covariates in our analyses.

In summary, VL and HL are highly prevalent among older adults and their co-occurrence may compound their respective impacts on health and functioning, thereby exerting strong effects on the mental health and wellbeing of those affected. Visual cues can provide an important compensatory mechanism for hearing impaired adults, and, conversely, auditory cues for adults with visual impairment. Our findings indicate that VL alone (in the absence of HL) has little effect on depressive symptoms, but does accentuate the effects of HL. Importantly, the association was explained by levels of participation in social activities and daily functioning.

The principal contribution of audiometric HL to increased depressive symptoms suggests that hearing assistive technologies and hearing rehabilitation programs may help to reduce the impacts of DSL. This evidence base can inform health policy which is needed to encourage improved awareness and screening of sensory loss and co-morbidities among older adults. There is a need for health professionals and care practitioners to be sensitive to the combined effects of vision and HL on mental health and wellbeing. Our findings point to the important roles that functional independence and activity engagement can play in mitigating these adverse impacts.

## Conflict of Interest Statement

The authors declare that the research was conducted in the absence of any commercial or financial relationships that could be construed as a potential conflict of interest.
